# Comparative performance of body roundness index and traditional obesity indices in predicting cardiovascular risk: machine learning insights from three prospective aging cohorts

**DOI:** 10.3389/fendo.2025.1653328

**Published:** 2025-09-25

**Authors:** Yinghuan Zhang, Yuxuan Wang, Shan Qiao, Xue Yang, Meihui Zhang, Chen Xu, Ying Wang, Fan Hu, Yong Cai

**Affiliations:** ^1^ School of Public Health, Shanghai Jiao Tong University, Shanghai, China; ^2^ Public Health Research Center, Tongren Hospital, Shanghai Jiao Tong University School of Medicine, Shanghai, China; ^3^ Department of Health Promotion, Education and Behavior, Arnold School of Public Health, University of South Carolina States, Columbia, SC, United States; ^4^ JC School of Public Health and Primary Care, Faculty of Medicine, The Chinese University of Hong Kong, Hong Kong, Hong Kong SAR, China; ^5^ Center for Community Health Care, China Hospital Development Institute, Shanghai Jiao Tong University, Shanghai, China

**Keywords:** cardiovascular diseases, obesity, prediction model, model validation, body roundness index

## Abstract

**Objective:**

The burden of cardiovascular diseases (CVD) is significant, necessitating early prevention, with obesity standing out as a pivotal modifiable risk factor. We aimed to use three prospective aging cohorts to develop an obesity-focused prediction model for incident CVD risk with enhanced validation and explanation.

**Methods:**

We analyzed longitudinal data from the China Health and Retirement Longitudinal Study (CHARLS) wave 1-4, Health and Retirement Study (HRS) wave 11-14, and English Longitudinal Study of Ageing (ELSA) wave 6-9. All participants were aged 45 years or older, had no CVD at baseline, and completed follow-up assessments across three subsequent waves. The main outcome was the occurrence of CVD (self-reported physician diagnoses of either heart disease or stroke). The predictors were screened by the Least Absolute Shrinkage and Selection Operator and Random Survival Forest. A multivariate Cox regression analysis was applied to develop the prediction model. Model performance was validated using: (1) concordance index for discrimination, (2) calibration curves for risk accuracy, and (3) time-dependent Receiver Operating Characteristic curves for classification. The time-dependent feature importance plot, partial dependence survival profiles and SHapley Additive exPlanations plot were used to interpret the model.

**Results:**

The study included 5768 participants from CHARLS, 3151 from HRS and 3016 from ELSA. The CVD incidence rates of CHARLS, HRS and ELSA were 21.2%, 13.2% and 13.5% respectively. Three of the seventeen screened covariates, which were age, hypertension, systolic blood pressure (SBP), as well as body mass index (BMI) and body roundness index (BRI), were included in the prediction model. The model exhibited a valid predictive value and moderate performance, with obesity showing a pronounced effect. BRI demonstrated stronger associations with CVD than BMI in both training and validation cohorts.

**Conclusion:**

Age, hypertension, SBP, BMI, and BRI were significant predictors of incident CVD in middle-aged and older adults, highlighting the impact of obesity on CVD risk, and consequently offered a valuable model for public health strategies to prevent CVD.

## 1 Introduction

Cardiovascular diseases (CVD) remain the leading cause of global mortality and a major contributor to disability worldwide (1). According to the Global Burden of Disease Study reports, the prevalence of total CVD cases nearly doubled from 1990 to 2019 (1), with CVD accounting for approximately 19.8 million deaths globally in 2022 (2). Extensive epidemiological studies have identified primary risk factors for CVDs, including age, sex, smoking status, alcohol consumption, hypertension, diabetes mellitus, obesity, and family history (3–6).

Among these risk factors, obesity emerges as a significant independent and modifiable risk factor for CVD (7–9). A study based on the Swedish twin population demonstrated that obesity was associated with CVD regardless of genetic or environmental predisposition (10). Furthermore, findings from the China Kadoorie Biobank cohort study indicate that obesity remains a risk factor for CVD independent of major metabolic factors among Chinese adults, suggesting that even metabolically healthy obese individuals exhibit increased risks for major vascular events (11). Notably, compared to general obesity as measured by Body Mass Index (BMI), abdominal obesity has been demonstrated to be a substantially stronger predictor of CVD risk (12). Various indices reflecting abdominal obesity and visceral fat distribution have been developed, including the Body Roundness Index (BRI) and A Body Shape Index (ABSI), which some studies suggest may have superior predictive capabilities compared to BMI (13, 14).

Following the seminal Framingham Heart Study, CVD risk assessment researches have evolved continuously, with models tailored to different countries and ethnic groups, including the ASCVD score (15), the SCORE2 model (16), and the QRISK3 model (17). Methodological approaches in model construction have also advanced significantly, progressing from conventional logistic regression to sophisticated machine learning techniques, exemplified by the neural network model for heart attack prediction developed by Maryam’s team (18). Among machine learning models handling right-censored survival data, Random Survival Forest (RSF), Survival Gradient Boosting, and Penalized Cox regression (including LASSO-Cox) represent the most widely adopted approaches in contemporary research (19).

Nevertheless, several limitations persist in current predictive models. Given the disparities in CVD profiles across nations, variations in behavioral and lifestyle patterns among populations, and genetic distinctions among ethnic groups, predictive models developed from single cohorts may not be universally applicable to diverse populations. Moreover, many algorithmic models, particularly those employing machine learning techniques, function as “black box” systems, lacking sufficient interpretability and making it challenging to fully elucidate their decision-making processes and outcomes (20, 21).

In the present study, we utilize data from three prospective aging cohorts: the China Health and Retirement Longitudinal Study (CHARLS), the Health and Retirement Study (HRS), and the English Longitudinal Study of Ageing (ELSA). The study aims to construct a comprehensive prediction model for incident CVD risk based on obesity indices, while enhancing the transparency and credibility of the machine learning approach through detailed model validation and interpretation.

## 2 Materials and methods

### 2.1 Study design and population

The CHARLS, HRS, and ELSA were all prospective and nationally representative cohorts conducted in China, the United States, and the United Kingdom, respectively. The present study utilized longitudinal data spanning wave 1 (2011) to wave 4 (2018) of CHARLS, wave 11 (2012) to wave 14 (2018) of HRS, and wave 6 (2012) to wave 9 (2018) of ELSA.

**Figure 1:**
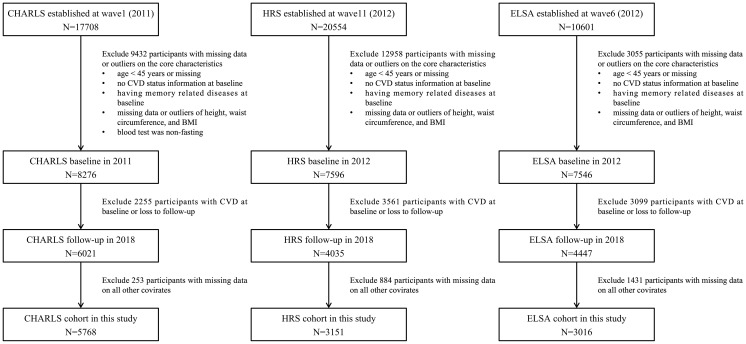
Selection process of the study population.

The inclusion criteria for the present study were: 1) age ≥45 years at baseline; 2) absence of heart disease and/or stroke at baseline. Exclusion criteria encompassed: 1) missing baseline data pertaining to age, CVD status, or essential covariates; 2) missing values or statistical outliers in anthropometric measurements (height, weight, waist circumference); 3) presence of memory-related diseases at baseline; 4) loss to follow-up; 5) non-fasting status during blood sample collection. The final analytical cohort comprised 5758 participants from CHARLS, 3151 from HRS, and 3016 from ELSA, all of whom had no CVD at baseline and completed follow-up assessments across three subsequent waves. The systematic participant selection process is detailed in **Figure 1**.

### 2.2 Calculation of obesity indices

This investigation incorporated five obesity indices as main exposure: BMI, BRI, ABSI, Waist-to-Height Ratio (WHtR), and Conicity Index (CI). These standardized indices were computed using physical measurements obtained under strictly controlled conditions, all of which adhered to standardized protocols, with height recorded in meters, weight in kilograms, and waist circumference in centimeters. The measurements were conducted by trained research personnel using calibrated equipment to ensure precision and reliability. Each anthropometric parameter was measured twice, with a third measurement performed if the difference between the first two exceeded predetermined thresholds, and the mean value was used for subsequent analyses. The specific calculation formulas are as follows.


$$\text{BMI}=\frac{\text{Weight}(\text{kg})}{\text{Height}{\left(\text{m}\right)}^{2}}$$

$$\text{BRI}=364.2-365.5\sqrt{1-(\frac{{\left(\text{WC}(\text{m})/2\text{π}\right)}^{2}}{{\left(0.5\times \text{Height}(\text{m})\right)}^{2}})}$$

$$\text{WHtR}=\frac{\text{WC}(\text{cm})}{\text{Height}(\text{cm})}$$

$$\text{CI}=\frac{\text{WC}(\text{m})}{0.019\sqrt{\frac{\text{Weight}(\text{kg})}{\text{Height}(\text{m})}}}$$

$$\text{ABSI}=\frac{\text{WC}}{{\text{Height}}^{1/2}\times {\text{BMI}}^{2/3}}$$

### 2.3 Assessment of CVD events

The primary outcome for this study was incident CVD, which was systematically ascertained through self-reported physician diagnoses of either heart disease or stroke across all three cohorts. Participants were specifically queried about receiving formal medical diagnoses of heart disease or stroke from qualified healthcare professionals. Incident cases were defined as participants who reported a new diagnosis of either condition during the follow-up period. The survival time was precisely quantified in months, with distinct calculations for two scenarios: for participants who developed CVD, the duration was measured from the baseline interview date to the follow-up interview date when the CVD event was first reported; for participants who remained CVD-free, the survival time was calculated from the baseline interview date through the final follow-up interview date.

### 2.4 Covariates

Baseline data collection was conducted by certified interviewers using standardized questionnaires. Socio-demographic variables encompassed age, sex, education level (less than lower secondary, upper secondary & vocational training, and tertiary), and marital status (married or other partnership status). Behavioral characteristics included physical activity (engaging in light, moderate or vigorous activities weekly), social activity (yes or no), smoking and drinking status (ever or never). Medical history documentation included hypertension, diabetes and cancer. Physical measurements included systolic blood pressure (SBP) and resting pulse rate. Laboratory assessments included glycated hemoglobin (HbA1c), high-density lipoprotein cholesterol (HDL-C), total cholesterol (TC) and C-reactive protein (CRP).

### 2.5 Statistical analysis

Comprehensive statistical analyses were performed using R software version 4.3.1. For descriptive statistics, continuous variables were expressed as median [interquartile range (IQR)], while categorical variables were expressed as frequency (percentage). Initial variable screening employed univariate Cox regression analysis. The analytical framework utilized CHARLS as the training set, HRS as the testing set, and ELSA as the external validation set. Variable selection implemented a dual-methodology approach combining the Least Absolute Shrinkage and Selection Operator for Cox Proportional Hazards Model (LASSO-Cox) and RSF algorithms on the training set. LASSO-Cox was performed using 10-fold cross-validation with the lambda 1-standard error (1-SE) criterion. RSF was implemented with 1000 trees (ntree = 1000), a minimum node size of 3 (nodesize = 3), and 5 randomly selected candidate variables per split (mtry = 5). Final predictor covariates were determined through the intersection of variables identified at lambda 1-SE in LASSO regression with the top 30% important variables identified by the RSF algorithm.

The prediction model was developed using multivariate Cox regression analysis. Model performance evaluation encompassed multiple dimensions: discrimination was assessed using the concordance index (C-index) across training, testing, and validation datasets; calibration was evaluated through calibration curve plots comparing predicted versus observed risks; and classification ability was determined using time-dependent receiver operating characteristic (ROC) curves with corresponding areas under the curves (AUC).

In addition, we evaluated the model interpretability using explainable machine learning methods with the “survex” R package (22). Time-dependent feature importance as a change in the loss function after variable value permutations based on the cumulative/dynamic (C/D) AUC were created for the model. To explore the complex relationship between variable values and time, partial dependence survival profiles (PDP) were created for the model. Time-dependent SHapley Additive exPlanations (SHAP) values for a single participant were also calculated to explain the contribution of each feature to the model’s predictions.

## 3 Results

### 3.1 Baseline characteristics of the study population

According to inclusion and exclusion criteria, 5768 participants from CHARLS (median age: 57 years, female: 54.2%), 3151 from HRS (median age: 62 years, female: 61.0%) and 3016 from ELSA (median age: 64 years, female: 54.8%) were included. The specific socio-demographic, behavioral, and health-related characteristics of the three cohorts at baseline are presented in **Table 1**.

**Table 1:** Baseline characteristics of participants in three cohorts.

Characteristics	CHARLS (N = 5768)	HRS (N = 3151)	ELSA (N = 3016)
Age (years)	57.00 [51.00, 63.00]	62.00 [56.00, 72.00]	64.00 [59.00, 70.00]
Sex			
Male	2641 (45.8)	1230 (39.0)	1364 (45.2)
Female	3127 (54.2)	1921 (61.0)	1652 (54.8)
Education level			
Less than lower secondary	5230 (90.7)	511 (16.2)	666 (22.1)
Upper secondary & vocational training	492 (8.5)	1818 (57.7)	1652 (54.8)
Tertiary	46 (0.8)	822 (26.1)	698 (23.1)
Marital status			
Married	4935 (85.6)	1958 (62.1)	2150 (71.3)
Other partnership status	833 (14.4)	1193 (37.9)	866 (28.7)
Physical activity			
No	247 (4.3)	68 (2.2)	63 (2.1)
Light	496 (8.6)	296 (9.4)	166 (5.5)
Moderate	737 (12.8)	1063 (33.7)	1291 (42.8)
Vigorous	1009 (17.5)	1724 (54.7)	1496 (49.6)
Missing	3279 (56.8)	–	–
Social activity			
No	3098 (53.7)	412 (13.1)	1745 (57.9)
Yes	2647 (45.9)	2179 (69.2)	1083 (35.9)
Missing	23 (0.4)	560 (17.8)	188 (6.2)
Smoking			
No	3571 (61.9)	1504 (47.7)	1231 (40.8)
Yes	2197 (38.1)	1647 (52.3)	1785 (59.2)
Drinking			
No	3502 (60.7)	1209 (38.4)	268 (8.9)
Yes	2266 (39.3)	1942 (61.6)	2748 (91.1)
Hypertension			
No	4428 (76.8)	1515 (48.1)	1940 (64.3)
Yes	1340 (23.2)	1636 (51.9)	1076 (35.7)
Diabetes			
No	5465 (94.7)	2572 (81.6)	2794 (92.6)
Yes	303 (5.3)	579 (18.4)	222 (7.4)
Cancer			
No	5734 (99.4)	2811 (89.2)	2756 (91.4)
Yes	34 (0.6)	340 (10.8)	260 (8.6)
Systolic Blood Pressure (mmHg)	125.00 [113.00, 139.50]	125.50 [115.00, 139.00]	130.50 [120.00, 142.00]
Pulse Rate (bpm)	71.50 [65.00, 78.00]	69.50 [62.50, 76.75]	65.50 [59.00, 72.00]
Glycated Hemoglobin (%)	5.10 [4.90, 5.40]	5.40 [5.20, 5.70]	5.72 [5.54, 5.99]
High-density Lipoprotein Cholesterol (mg/dL)	49.87 [40.98, 60.31]	85.00 [71.00, 99.00]	61.76 [50.18, 77.20]
Total Cholesterol (mg/dL)	191.37 [168.56, 216.11]	304.00 [265.00, 348.50]	220.02 [189.14, 247.04]
C-reactive protein (mg/L)	0.97 [0.53, 2.01]	1.98 [0.91, 4.19]	1.40 [0.70, 2.90]
Body Mass Index	23.11 [20.89, 25.66]	28.95 [25.54, 33.14]	27.30 [24.75, 30.44]
Body Roundness Index	4.01 [3.21, 5.04]	5.45 [4.29, 6.82]	4.72 [3.77, 5.88]
Waist-to-Height Ratio	0.53 [0.49, 0.58]	0.60 [0.55, 0.66]	0.57 [0.52, 0.62]
Conicity Index	1.28 [1.23, 1.34]	1.32 [1.26, 1.37]	1.29 [1.22, 1.34]
A Body Shape Index	0.08 [0.08, 0.09]	0.08 [0.08, 0.09]	0.08 [0.08, 0.08]

Continuous variables are expressed as median [IQR]; categorical variables are expressed as frequency (percentage).

A total of 2053 participants (1227 from CHARLS, 417 from HRS, and 409 from ELSA) developed CVD during follow-up, and the incidence rates were 21.2%, 13.2% and 13.5% respectively. The Kaplan-Meier survival curves for incident CVD in three cohorts are illustrated in **Supplementary Figure 1**.

Associations of baseline characteristics with risks of incident CVD are shown in the **Supplementary Table 1**. The significantly elevated risks of incident CVD were found in participants with higher obesity indices in three cohorts.

### 3.2 Prediction model construction

**Figure 2:**
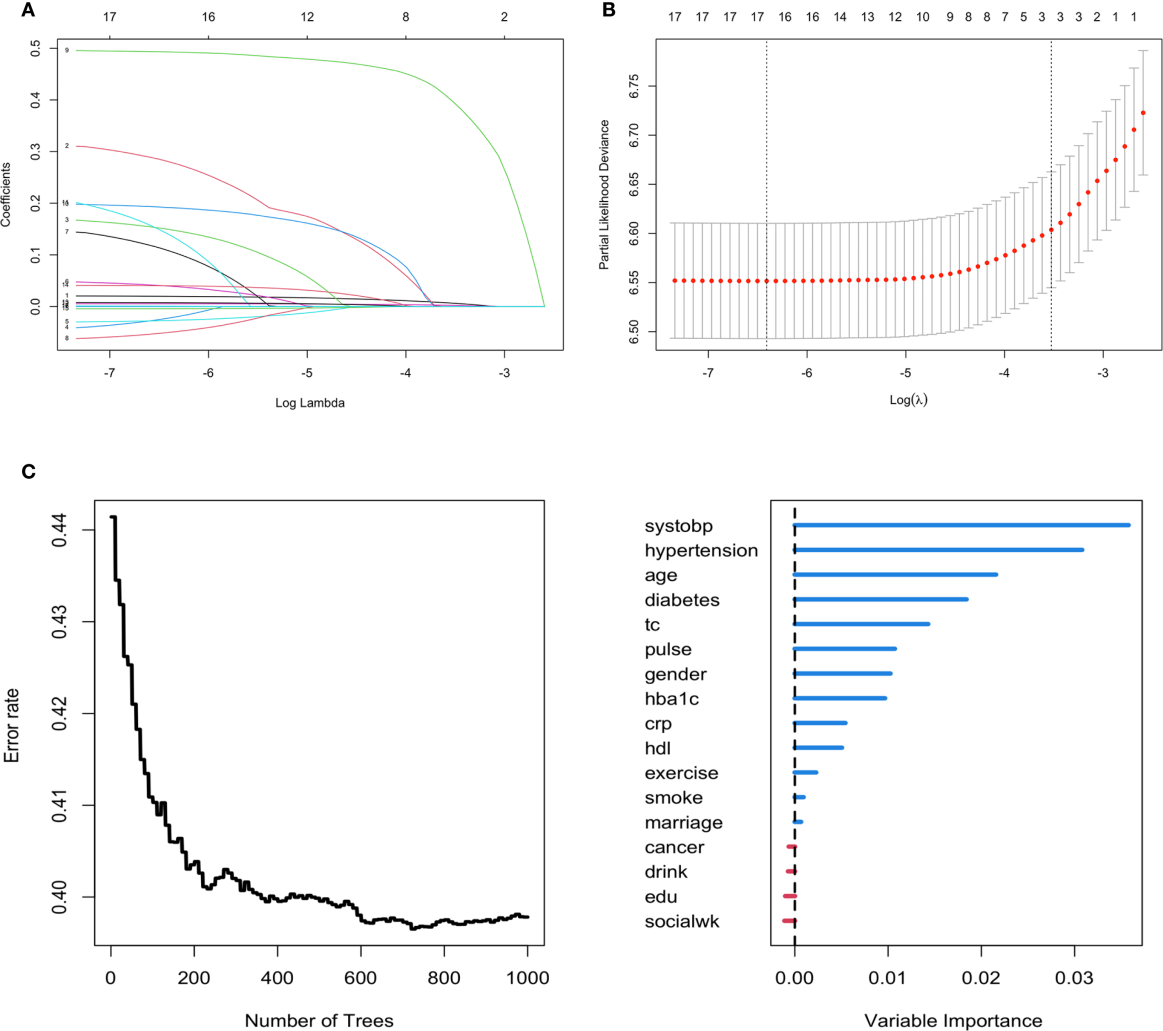
Feature selection using the LASSO-Cox regression and RSF model for incident CVD risk. **(A, B)** Variable selection by the LASSO Cox regression model. **(C)** Feature selection using the RSF model.

The features with nonzero coefficients selected by LASSO-Cox regression included age, hypertension, and SBP. The lambda 1-SE of these indicators were 0.007, 0.397, and 0.002 respectively (**Figures 2A, B**). Apart from social activity, drinking, education level and cancer, the remaining 13 covariates were selected by the RSF algorithm. Among these, the covariates deemed most significant, falling within the top 30% of variable importance, comprised SBP, hypertension, age, and diabetes. The variable importance of these indicators was 0.035, 0.031, 0.022 and 0.019 respectively (**Figure 2C**). The intersection of variables selected by the two algorithms resulted in the following covariates being included in the prediction model: age, hypertension, and SBP. These selected significant factors were included in the multivariate Cox regression analysis to construct the prediction model for incident CVD risk. The coefficients are shown in **Table 2**.

**Table 2:** Multivariate cox regression analysis of the predictors for incident CVD risk.

Variables	Z	Hazard ratio	95% CI	*P*-value
Age	5.800	1.020	1.013-1.027	<0.001^a^
Hypertension	7.578	1.665	1.459-1.900	<0.001^a^
Systolic Blood Pressure	2.990	1.004	1.002-1.007	0.003^a^
Body Mass Index	1.396	1.016	0.994-1.038	0.163
Body Roundness Index	2.689	1.084	1.022-1.150	0.007^a^

Data presented as the Z, Hazard Ratio and 95%CI. Statistical significance recognized as *P* < 0.05 and denoted by ^a^.

### 3.3 Prediction model validation

**Figure 3:**
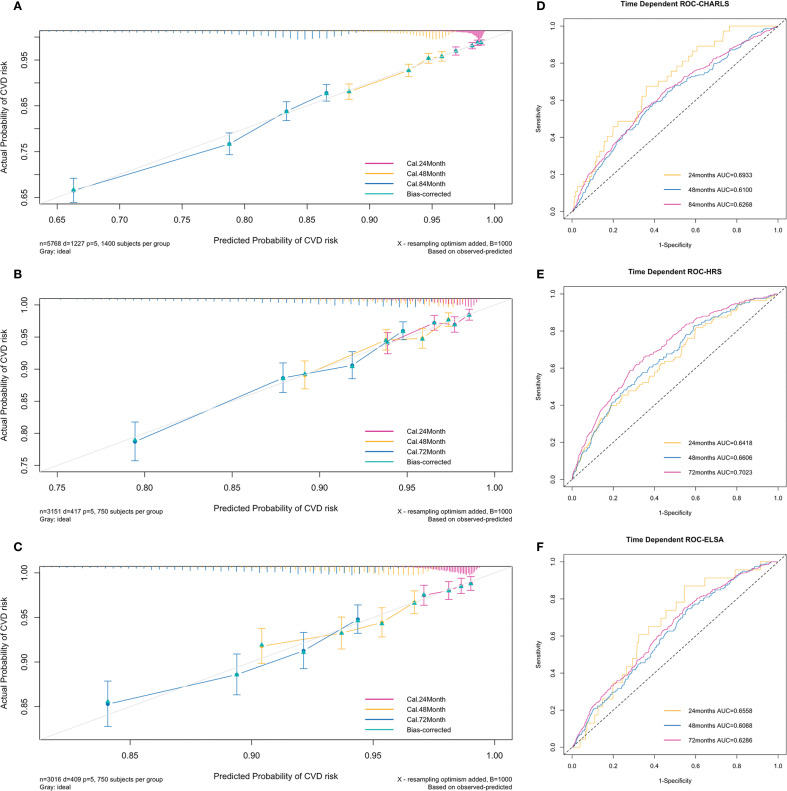
Calibration plots and time-dependent ROC curves of the incident CVD risk model. **(A–C)** Calibration plots comparing predicted and actual survival probabilities of the incident CVD risk model for training **(A)**, testing **(B)** and validation set **(C)**. The y-axis represents actual probability of CVD risk, the x-axis represents the predicted probability of CVD risk, and the grey diagonal line represents the reference line. **(D–F)** Time-dependent ROC curves of the model for training **(D)**, testing **(E)** and validation set **(F)**. The y-axis represents the true positive rate of the prediction, and the x-axis represents the false positive rate of the prediction.

The C-index of the prediction model was 0.63 in the training set, 0.663 in the testing set, and 0.621 in the validation set. **Figures 3A–C** presents the 1000-sample bootstrapped calibration plot for the incident CVD prediction model across the training, testing and validation sets. The calibration plots revealed an excellent agreement between the predicted and actual risks, illustrating the model’s good predictive accuracy.

The time-dependent ROC curves to predict incident CVD at three follow-up waves are plotted in **Figure 3**. The AUCs at 24, 48, and 84 months in the training set were 0.69 (95% CI = 0.62-0.77), 0.61 (95% CI = 0.57-0.65), and 0.63 (95% CI = 0.60-0.65) respectively (**Figure 3D**). The AUCs at 24, 48, and 72 months in the testing set were 0.64 (95% CI = 0.58-0.70), 0.66 (95% CI = 0.62-0.70), and 0.70 (95% CI = 0.67-0.73) respectively (**Figure 3E**). The AUCs at 24, 48, and 72 months in the validation set were 0.66 (95% CI = 0.56-0.75), 0.61 (95% CI = 0.57-0.65), and 0.63 (95% CI = 0.60-0.66) respectively (**Figure 3F**). The results indicate a valid predictive value and moderate model performance.

### 3.4 Prediction model explanation

**Figure 4:**
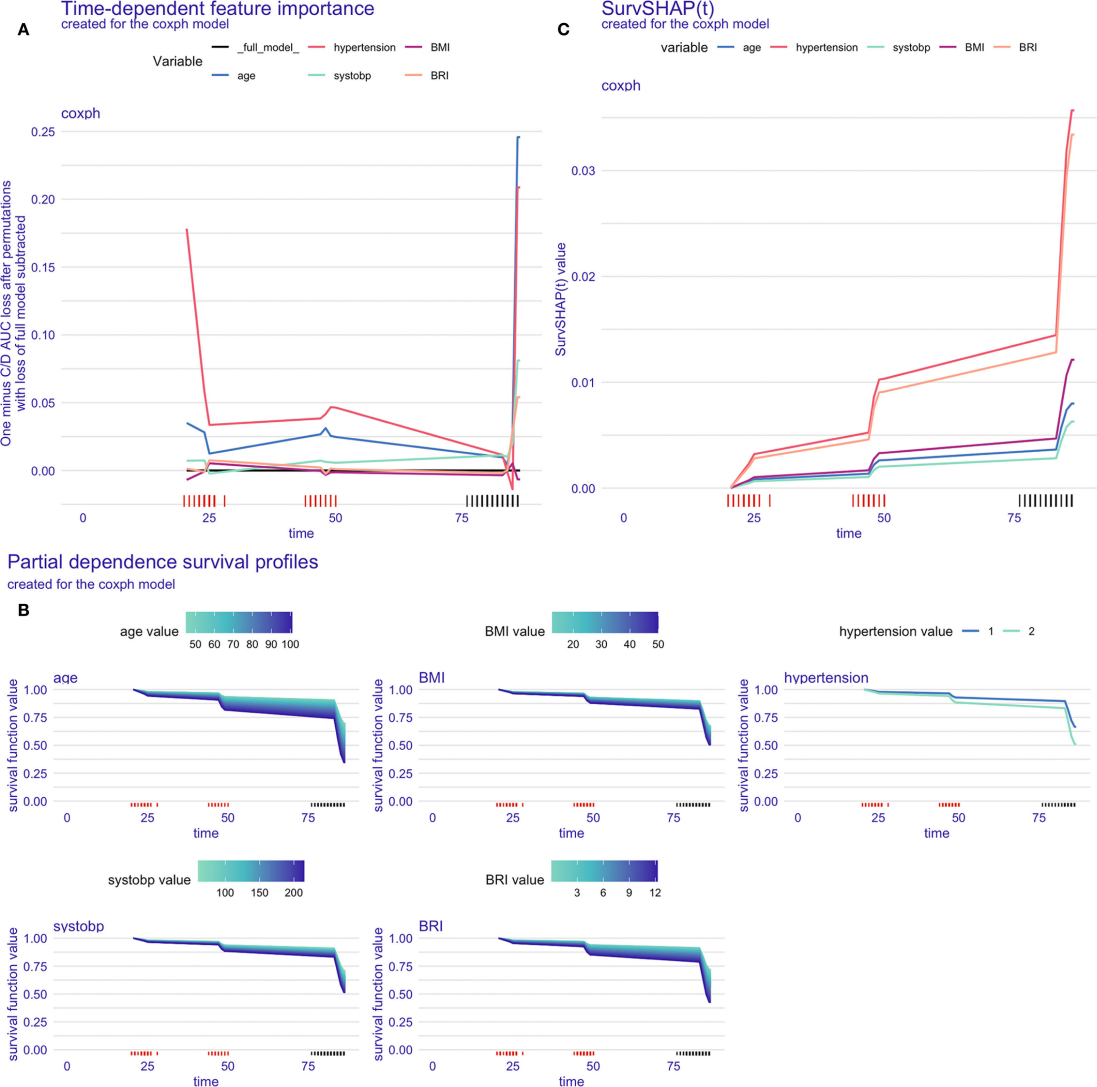
Model interpretation in the training set. **(A)** Time-dependent feature importance of the model, C/D AUC loss after permutation; **(B)** Time-dependent partial dependence survival profile of the model; **(C)** SurvSHAP(t) plot for a single participant.

**Figure 4** presents the model interpretability results within the training set (CHARLS). In the feature importance analysis based on C/D AUC, variables that induced a greater increase in the loss function had a more significant impact on the incidence of CVD. As shown in **Figure 4A**, hypertension remained at a relatively higher position throughout the entire follow-up period, followed by age, indicating that these two variables are of greater importance in the CVD onset.

The PDP illustrated the marginal effects of different features on the model’s predictive outcomes, showing how the outcome would change if the value of one feature is altered while keeping all other features constant. According to **Figure 4B**, the influence of age and BRI on the incidence of CVD was stronger than that of other variables, as indicated by the wider distribution bands, which suggest a larger probability of different CVD outcomes among participants with varying values.

**Figure 4C** displayed the time-dependent survival SHAP plot for a randomly selected participant. Positive SHAP values were indicative of a higher CVD risk. An increase in the values of all five variables was associated with an increased risk of CVD incidence, with hypertension and BRI demonstrating the most pronounced effects.

The model explanation results for the testing set (HRS) and the validation set (ELSA) were presented in the **Supplementary Figure 2** and **Supplementary Figure 3**.

## 4 Discussion

In this study, we implemented advanced survival machine learning algorithms to develop an obesity-focused predictive model for incident CVD risk in middle-aged and older populations. The model was constructed using longitudinal data from three large-scale prospective population-based cohorts: CHARLS, HRS, and ELSA. Our analyses revealed that age, hypertension, SBP, BMI and BRI emerged as statistically significant correlates and robust predictors of CVD incidence.

Consistent with our research hypothesis, obesity demonstrated substantial predictive significance for CVD occurrence, with this association maintaining robustness following adjustment for other potent correlates. Obesity is well-established as an independent risk factor for CVD, supported by extensive epidemiological evidence. Meanwhile, obesity can also contribute to the development of CVD through its associations with traditional and non-traditional CVD risk factors. The metabolic syndrome, of which central obesity constitutes an important component, is strongly associated with CVD development (23). The association between obesity and CVD may be influenced by potential confounders, such as dietary conditions (24), physical activity, and early-life factors (25). Notably, certain individuals with obesity exhibit a metabolically healthy phenotype, which may be attributed to the protective role of brown adipose tissue (26). Future research should further elucidate the underlying mechanisms linking obesity to CVD.

It is proposed that obesity, traditionally defined as an excess of body fat that causes adverse effects on health, can no longer be measured solely by BMI (expressed in kg/m²) (27). Extensive investigation of body composition has led to a growing consensus that visceral fat poses greater health risks than subcutaneous fat. Emerging obesity indices such as BRI and WHtR include additional measurements beyond height and weight, specifically waist circumference, thereby providing more precise assessments of central obesity and abdominal fat distribution (28). Our final predictive model incorporated both BMI and BRI as complementary obesity indicators. While BRI showed higher coefficient values, reflecting the added predictive significance of central obesity measures for CVD risk in middle-aged and older adults, further validation is needed to determine its generalizability across age and ethnic groups. Although indices such as ABSI showed significance in univariate regression analyses, their performance diminished in multivariate models. This phenomenon may be attributed to their relatively compressed numerical scale, resulting in reduced overall variability, consequently leading to their associations being attenuated by stronger predictors in multivariate regression analyses.

Age also serves as a significant predictor for the CVD incidence, demonstrating a positive correlation between advanced age and elevated CVD risk. The Coronary Artery Risk Development in Young Adults Study illustrated the life course of cardiovascular health. Beginning in middle age (about 45 years), populations face moderate risk, with 20%-40% exhibiting subclinical disease manifestations. In the older cohort (≥65 years), risk elevation becomes pronounced, with 60%-80% demonstrating subclinical disease and approximately 15% experiencing cardiovascular events (29). Research indicated that the overall prevalence rate of stroke among individuals aged 60 years and older reaches 7.6%. Age-stratified analyses revealed that the risk of stroke for those aged 75–84 years is 2.4 times higher than the 60–74 age group, with individuals aged 85 years and above demonstrating a threefold increased risk (30).

Hypertension has been consistently identified as a primary risk factor and predictor for CVD in numerous studies. The Framingham Heart Study initially demonstrated through longitudinal cohort data that hypertension is a major risk factor for CVD (31). A study based on the Kailuan cohort in China also indicated the significant association between hypertension and CVD events. Following adjustment for other established CVD risk factors, the hazard ratio was 1.67 (95% CI: 1.28-2.17) for total CVD events in the hypertension group (32). Findings from the Atherosclerosis Risk in Communities study showed that standing SBP ≥140 mmHg was significantly associated with elevated CVD risk (33). In the present study, both self-reported hypertension history and measured SBP emerged as significant predictors of incident CVD. We contend that the concurrent inclusion of these two variables holds particular clinical relevance and methodological validity, as individuals with documented hypertension may exhibit either well-controlled blood pressure through medical interventions or suboptimal pressure control.

The model developed in this study demonstrated a moderate level of predictive ability and performance. In studies investigating the impact of obesity on CVD risk, previous research has utilized the Net Reclassification Index to evaluate the changes in predictive ability of a model after incorporating obesity (34). Similarly, another study has added central obesity, represented by waist circumference and waist-to-hip ratio, to the traditional Framingham model and found that central obesity makes a significant and independent contribution to cardiovascular outcomes (35). Our study employed a predictive modelling framework to examine the determinants of CVD incidence, and subsequently applied interpretable machine learning techniques to elucidate the influence of these predictors on the outcome, as well as to delineate their temporal variations.

Furthermore, this study identified cumulative incidence rates of CVD across three large population-based cohorts, which were 21.2% in China, 13.2% in the US, and 13.5% in the UK. These disparities may be partially explained by racial and ethnic variations (36) or genomic diversity (37). However, these differences more likely reflect disparities in socioeconomic development and lifestyle changes. The harmonized follow-up period (2011/2012-2018) coincided with China’s rapid economic expansion and elevated living standards, potentially contributing to increased CVD risk factor prevalence, including obesity and chronic diseases, thereby elevating CVD incidence. Beyond this, systemic differences in healthcare delivery may contribute to the observed incidence disparities. Compared with the US and UK, China faces challenges in primary care service capacity and the implementation of tiered diagnosis systems (38, 39), which may affect CVD prevention and early detection. Moreover, while international diagnostic guidelines are widely adopted, their implementation consistency may vary across clinical settings, which may lead to underdiagnosis or misclassification.

Several limitations warrant careful consideration in interpreting our findings. Firstly, as an analysis utilizing questionnaire data from cohort studies, this study was inevitably affected by missing data and potential recall bias, particularly concerning self-reported CVD diagnosis and medical history. Secondly, the restricted coverage of the databases allowed for the inclusion of only 17 covariates in our analysis, potentially omitting important predictors such as genetic factors, family history, detailed dietary patterns, environmental exposures, and comprehensive socioeconomic indicators. Methodologically, our study may have been affected by survival bias in the older population, and we were unable to account for time-varying covariates during the follow-up period. The dynamic nature of obesity indices over time could not be fully captured, and residual confounding may persist despite statistical adjustment. The model-specific limitations include potential challenges in capturing non-linear relationships between predictors and outcomes, and limited validation of prediction performance in specific subgroups. The follow-up period (2011/2012-2018) may be insufficient for capturing long-term CVD outcomes, and temporal changes in healthcare systems and treatment patterns could not be accounted for. Future research should address these limitations through the incorporation of additional relevant predictors, more sophisticated handling of missing data, extended follow-up periods, integration of time-varying covariates, validation in more diverse populations, implementation of more advanced statistical methods for handling complex relationships, and evaluate the model’s clinical net benefit at different risk thresholds and time horizons.

## 5 Conclusions

This study demonstrates that age, hypertension, SBP, BMI, and BRI constitute meaningful predictors for incident CVD among middle-aged and older adults across Chinese, American, and British populations. Our findings provide new evidence supporting the longitudinal associations between these factors and CVD risk, particularly emphasizing the role of obesity. The findings also provide a theoretical basis for the potential application value of BRI in CVD risk prediction.

## Data Availability

Publicly available datasets were analyzed in this study. This data can be found here: Data of this study are available from official websites of China Health and Retirement Longitudinal Study (http://charls.pku.edu.cn), English Longitudinal Study of Ageing (https://ukdataservice.ac.uk), and Health and Retirement Study (https://hrsdata.isr.umich.edu).
