# Supplementary material for: Comparative performance of body roundness index and traditional obesity indices in predicting cardiovascular risk: machine learning insights from three prospective aging cohorts
Source: Front Endocrinol (Lausanne). 2025 Sep 25;16:1653328. doi: 10.3389/fendo.2025.1653328 (PMC12507581; doi:10.3389/fendo.2025.1653328)
Supplement: Supplementary file 1 [file DataSheet1.docx]

Supplementary Material

# Supplementary Figures


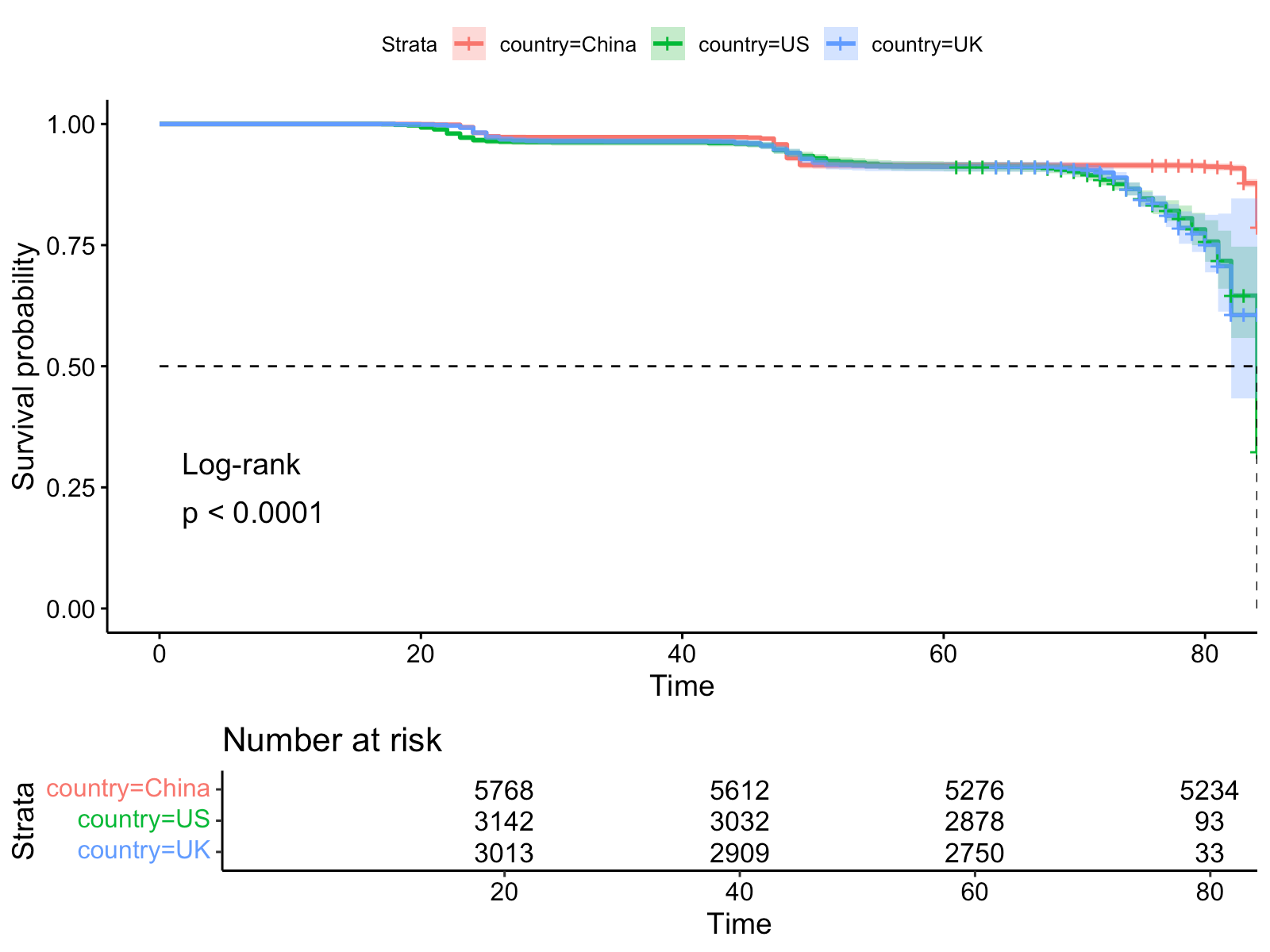


**Supplementary Figure 1.** Kaplan-Meier survival curves for incident CVD in three cohorts.

**
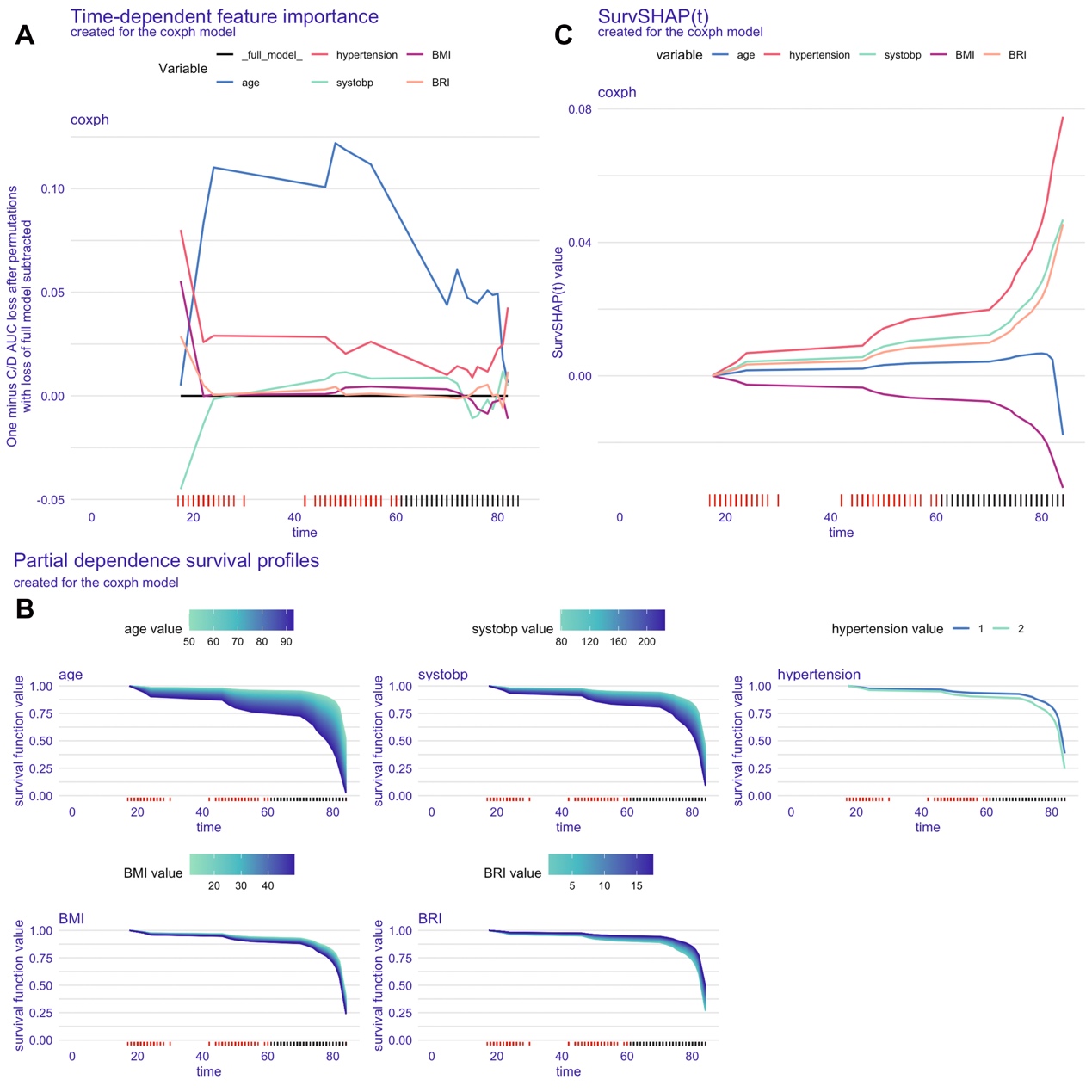
**

**Supplementary Figure 2.** Model interpretation in the testing set. **(A)** Time-dependent feature importance of the model, C/D AUC loss after permutation; **(B)** Time-dependent partial dependence survival profile of the model; **(C)** SurvSHAP(t) plot for a single participant.


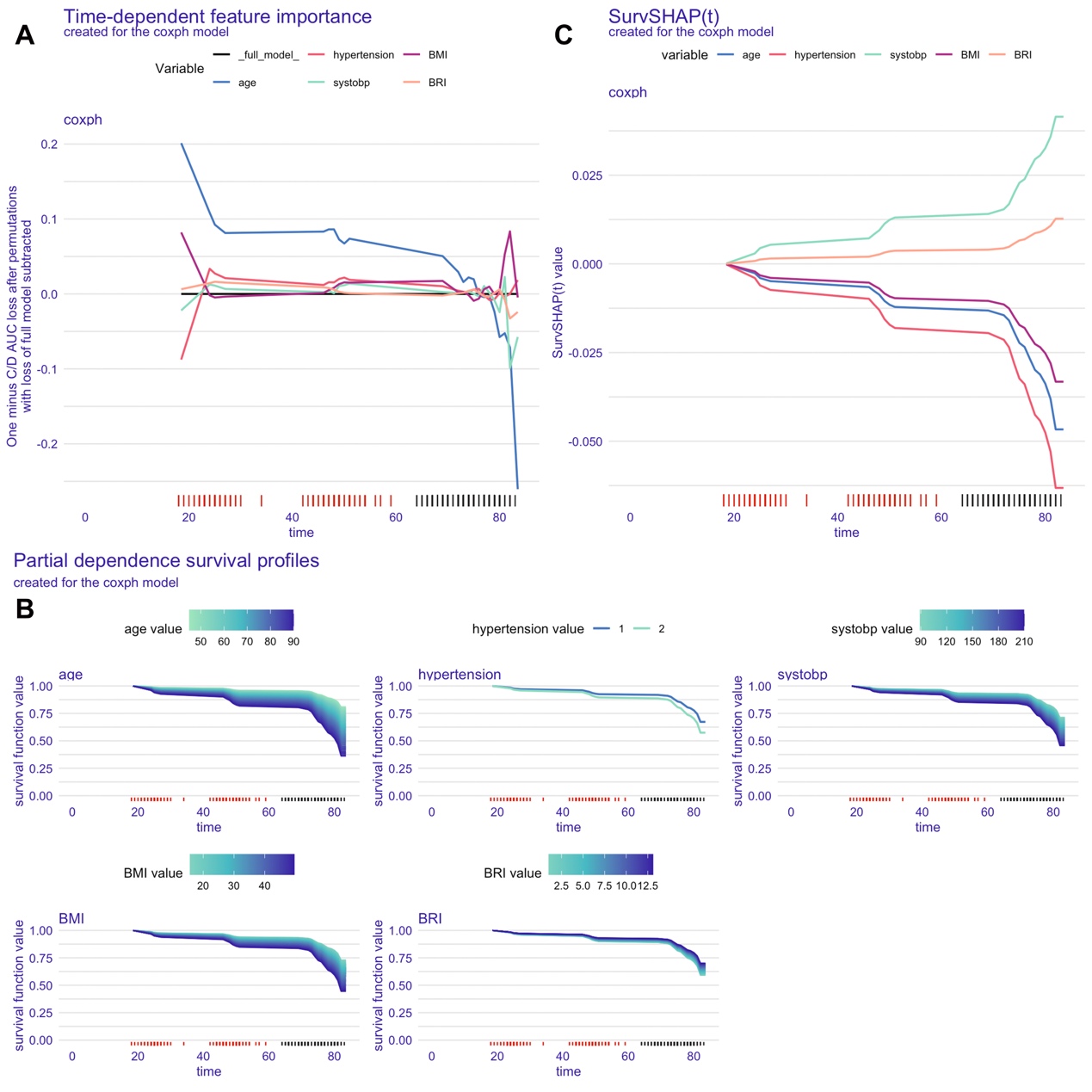


**Supplementary Figure 3.** Model interpretation in the validation set. **(A)** Time-dependent feature importance of the model, C/D AUC loss after permutation; **(B)** Time-dependent partial dependence survival profile of the model; **(C)** SurvSHAP(t) plot for a single participant.

# Supplementary Tables

**Supplementary Table 1.** Univariate Cox regression of the correlates for incident CVD risk

| **Characteristics** | **CHARLS (N=5768)**  **HR (95%CI)** | **HRS (N=3151)**  **HR (95%CI)** | **ELSA (N=3016)**  **HR (95%CI)** |
| --- | --- | --- | --- |
| Age (years) | 1.026 (1.020, 1.032) *** | 1.048 (1.038, 1.058) *** | 1.044 (1.032, 1.056) *** |
| Sex |  |  |  |
| Male | Ref | Ref | Ref |
| Female | 1.273 (1.136, 1.426) *** | 0.708 (0.584, 0.858) *** | 0.643 (0.529, 0.782) *** |
| Education Level |  |  |  |
| Less than lower secondary | Ref | Ref | Ref |
| Upper secondary & vocational training | 1.096 (0.902, 1.331) | 1.053 (0.801, 1.385) | 0.790 (0.626, 0.998) * |
| Tertiary | 1.016 (0.545, 1.894) | 1.057 (0.778, 1.437) | 0.859 (0.651, 1.133) |
| Marital Status |  |  |  |
| Married | Ref | Ref | Ref |
| Other partnership status | 1.136 (0.975, 1.325) | 1.124 (0.924, 1.367) | 0.980 (0.790, 1.215) |
| Physical Activity |  |  |  |
| No | Ref | Ref | Ref |
| Light | 0.938 (0.697, 1.263) | 0.791 (0.417, 1.500) | 1.643 (0.793, 3.405) |
| Moderate | 0.773 (0.580, 1.030) | 0.774 (0.430, 1.394) | 0.945 (0.484, 1.847) |
| Vigorous | 0.584 (0.440, 0.776) *** | 0.651 (0.364, 1.165) | 0.884 (0.453, 1.726) |
| Missing | 0.765 (0.594, 0.985) * | - | - |
| Social Activity |  |  |  |
| No | Ref | Ref | Ref |
| Yes | 1.054 (0.942, 1.179) | 0.809 (0.617, 1.059) | 1.013 (0.822, 1.248) |
| Missing | 1.479 (0.702, 3.114) | 0.810 (0.580, 1.133) | 1.508 (1.062, 2.143) * |
| Smoking |  |  |  |
| No | Ref | Ref | Ref |
| Yes | 0.900 (0.801, 1.012) | 1.215 (1.001, 1.475) * | 1.330 (1.085, 1.631) ** |
| Drinking |  |  |  |
| No | Ref | Ref | Ref |
| Yes | 0.873 (0.777, 0.981) * | 0.848 (0.698, 1.030) | 0.759 (0.559, 1.032) |
| Hypertension |  |  |  |
| No | Ref | Ref | Ref |
| Yes | 2.159 (1.923, 2.425) *** | 1.870 (1.527, 2.290) *** | 1.772 (1.460, 2.152) *** |
| Diabetes |  |  |  |
| No | Ref | Ref | Ref |
| Yes | 1.769 (1.444, 2.166) *** | 1.358 (1.081, 1.706) ** | 1.300 (0.931, 1.816) |
| Cancer |  |  |  |
| No | Ref | Ref | Ref |
| Yes | 1.167 (0.582, 2.339) | 1.249 (0.939, 1.663) | 1.494 (1.108, 2.013) ** |
| Systolic Blood Pressure  (mmHg) | 1.012 (1.009, 1.014) *** | 1.013 (1.008, 1.018) *** | 1.014 (1.008, 1.019) *** |
| Pulse rate (bpm) | 1.009 (1.004, 1.015) *** | 0.990 (0.981, 0.999) * | 1.004 (0.994, 1.013) |
| Glycated Hemoglobin (%) | 1.141 (1.077,1.210) *** | 1.211 (1.068, 1.372) ** | 1.144 (1.015, 1.290) * |
| High-density Lipoprotein  Cholesterol (mg/dL) | 0.993 (0.989, 0.997) *** | 0.997 (0.993, 1.001) | 0.991 (0.986,0.997) ** |
| Total Cholesterol (mg/dL) | 1.001 (1.000, 1.003) | 0.998 (0.997, 1.000) * | 0.997 (0.995, 1.000) * |
| C-reactive protein (mg/L) | 1.005 (0.999, 1.012) | 1.007 (0.998, 1.015) | 1.015 (1.007, 1.023) *** |
| Body Mass Index | 1.054 (1.040, 1.069) *** | 1.000 (0.983, 1.017) | 1.029 (1.010, 1.049) ** |
| Body Roundness Index | 1.216 (1.170, 1.263) *** | 1.024 (0.976, 1.073) | 1.104 (1.046, 1.165) *** |
| Waist-to-Height Ratio | 64.05 (27.84, 147.3) *** | 1.765 (0.568, 5.490) | 11.04 (3.224, 37.82) *** |
| Conicity Index | 11.88 (6.491, 21.73) *** | 4.732 (1.713, 13.07) ** | 9.785 (3.467, 27.61) *** |
| A Body Shape Index ^a^ | 1.297 (1.181, 1.425) *** | 1.300 (1.103, 1.531) ** | 1.390 (1.159, 1.668) *** |

**Notes:** ****P*<0.001, ***P*<0.01, **P*<0.05; ^a^ ABSI was amplified by a factor of 100 when included in the regression.
